# Consequences of COVID-19 Lockdown on Food Insecurity and Food Quality in Two Mediterranean Countries (Spain and Morocco)

**DOI:** 10.3390/foods14152604

**Published:** 2025-07-24

**Authors:** Rekia Belahsen, Mohamed Cherkaoui, Ana Isabel Mora Urda, Francisco Javier Martín Almena, María del Pilar Montero López

**Affiliations:** 1 Research Unit on Nutrition and Food Sciences, Laboratory of Anthropogenetic, Biotechnologies and Health, Department of Biology, Faculty of Sciences, Chouaib Doukkali University, El Jadida 24000, Morocco; 2 LPNAE Laboratoire de Pharmacologie, Neurobiologie, Anthropologie et Environnement SNDD, Stratégie Nationale de Développement Durable, Faculté de Sciences Semlalia, Université Cadi Ayyad de Marrakech, Marrakech 40000, Morocco; 3 Faculty of Teacher Training, Autonomous University of Madrid, 28049 Madrid, Spain; ana.mora@uam.es; 4 Area of Nutrition and Food Science, Department of Biomedical Sciences, University of Alcalá, 28871 Alcalá de Henares, Spain; franciscoj.martin@uah.es; 5 Department of Biology, Faculty of Sciences, Autonomous University of Madrid, 28049 Madrid, Spain

**Keywords:** food insecurity, lockdown, COVID-19, Spain, Morocco

## Abstract

Food security is defined as a state in which all people at all times have both physical and economic access to sufficient food to meet their dietary needs for a productive and healthy life. The general objective of this work was to assess the situation of food insecurity and its impact on the quantity and quality of food consumption during lockdown in the first wave of the COVID-19 pandemic and to identify the determinants associated with the different food insecurity (FI) categories on a sample of 2227 people (1168 people from Spain and 1059 people from Morocco). Food insecurity (FI) assessed by the Household Food Insecurity Access Scale (HFIAS) were compared in both countries, controlling for the effect of sociodemographic variables, age, gender, marital status, and education level. The mean HFIAS was 0.53 in the Spanish and 3.55 in the Moroccan samples (*p* < 0.001). Only 2.1% of the Spanish sample were in a situation of severe insecurity against 15.5% in Morocco (*p* < 0.001). Moroccans with moderate and severe food insecurity decreased their consumption of meat, fish, eggs, nuts, legumes, and fruits. The risk of food insecurity was higher in men than in women, in separated or divorced people, in people with secondary and middle education, and in younger people.

## 1. Introduction

Five years after the declaration of the COVID-19 pandemic, numerous scientific results derived from studies conducted at that time provide valuable information about its impact on the health, well-being, and behavior of the population. Some authors have also highlighted the risk of different pathogens generating new pandemics, the effects of which remain to be determined, and the response to vary from country to country, as was the case with COVID-19 [1,2,3]. Undoubtedly, access to food and changes in eating behaviors were some of the aspects that generated the greatest concern and interest and that will be of great importance in future pandemics. Furthermore, a sufficient intake of nutrients in the right proportions could help during the recovery process from the disease, especially in longer-term cases [4].

Food security (FS) continues to constitute a challenge not only for developing countries but also for the developed world [5,6,7]. Food is one of the areas of daily life that is affected during periods of crisis. The Household Food Insecurity Access Scale (HFIAS) defined food security as a situation in which all people at all times have physical and economic access to sufficient food to meet their dietary needs for a productive and healthy life [8,9,10]. According to the Food and Agriculture Organization (FAO), FS is the guarantee of regular, permanent, and free access, either directly or through monetary purchases, to quantitatively and qualitatively adequate and sufficient food corresponding to the cultural traditions to which the consumer belongs, and this guarantees a psychological, physical, individual, and collective life, without anxiety, and satisfying and dignifying [11].

This state includes feelings of uncertainty or anxiety over food (situation, resources, or supply; perceptions that food is of insufficient quantity (for adults and children); perceptions that food is of insufficient quality (includes aspects of dietary diversity, nutritional adequacy, and preference); reported reductions of food intake (for adults and children); and feelings of shame for resorting to socially unacceptable means to obtain food resources.

March 2025 marks 5 years since many countries implemented lockdowns to address the first wave of the COVID-19 pandemic. At the time this study was conducted, between April and June 2020, the COVID-19 pandemic was at a critical point in both Spain and Morocco. According to official sources, Spain had reported approximately 27,000 to 30,000 COVID-19-related deaths during this period, with excess mortality estimates reaching up to 44,000 deaths when accounting for underreporting and indirect effects [12,13]. In contrast, Morocco had registered 228 deaths by the end of June 2020, with 170 reported in April and 205 in May. These figures highlight the stark contrast in the pandemic’s impact between the two countries and help frame the context in which food access and household food insecurity were assessed in this study [14,15].

The consequences for access to food varied depending on the economic and social situations of the countries, both before and during the pandemic. Therefore, the impact on food insecurity also varied across countries and population groups within these countries.

Because of its contagious nature, the COVID-19 pandemic has become the most significant threat humanity has faced in this century [16]. To prevent the multiple risks posed by this health crisis, governments had adopted a series of measures. Among these, the restriction of the mobility of the population led to a stoppage of activities affecting Gross Domestic Product (GDP) in general and employment on a global scale [17] and affected access to certain foods, which had a significant impact on dietary behavior. These measures led to disruption of the food supply chain and the loss of income for many people, with different repercussions on household food security and different consequences depending on the economic development of each country or area [18,19,20,21,22]. According to the Committee on World Food Security, food security was compromised for large sectors of the population. The data presented by this committee were certainly alarming since they indicate that even before the COVID-19 pandemic, close to “2 billion people suffered from moderate or severe food insecurity” [23], to which it is estimated that between 83 and 132 million more people have joined as a direct consequence of this pandemic situation.

Access to food was different in the countries and was largely determined by the measures of the governments, both to guarantee the availability and distribution of food to the population, as well as those destined to protect the most disadvantaged groups that, in many cases, lost their jobs or saw their income drop. In Morocco, for example, health, economic, and social measures have been developed to cope and control [24]. However, a deceleration in the pace of growth in household consumption was observed with a loss of 589,000 jobs in the 2nd quarter of 2020 by the Moroccan economy, due to the spread of the COVID-19 pandemic, the state health emergency, and total lockdown [25]. Following the lockdown imposed by the State, the increase in the unemployment rate has in fact induced a reduction of 6.7% in household consumption expenditure in several areas, notably catering [26], with multiple repercussions on household access to a healthy and diversified diet [27]. For its part, the Spanish government launched a battery of measures aimed at reducing the impact of the crisis on households, workers, and firms. In addition, other measures were adopted to guarantee essential supplies of electricity, water, gas, and telecommunications to protect particularly vulnerable households. Adapted working hours and teleworking were facilitated whenever possible, and temporary layoffs (ERTE) were used so that employees could maintain their income and return to work once the lockdown ended [28].

At the beginning of the COVID-19 pandemic and during lockdown, the Agence Universitaire de la Francophonie funded the project ‘Analyse de la consommation alimentaire des familles en raison du lockdown suite à la pandémie de COVID-19 dans les pays du Nord et du Sud du Bassin Méditerranéen et son impact sur l’état nutritionnel’. DREO-AAP-COVID-n°2310-524968-Convention-A2-Madrid-721. The aim of that project was to determine the dietary behavior of the Mediterranean populations and to compare it with the dietary behavior prior to the pandemic and to lockdown, in two samples from the university environment (since the project was funded by a University Agency) belonging to two countries, Spain and Morocco, in the Mediterranean basin [29,30].

Additional information on food insecurity was also collected using the food insecurity index (FI) validated by the FAO. The results presented in this article come from this project.

The main hypothesis is that the lockdown during the first wave of the COVID-19 pandemic had a negative impact on food security, especially in populations with lower levels of social and economic protection, as is the case in Morocco compared to Spain.

The general objective of this work was to compare the degree of food insecurity during lockdown in the first wave of the COVID-19 pandemic in a sample made up of 1168 people from Spain and 1059 people from Morocco. The impact of the severity of food insecurity on the quantity and quality of food consumed is also compared, considering sociodemographic variables of both samples.

## 2. Material and Methods

It is a cross-sectional study, developed from 1 April to 30 June 2020, time of social distancing and restrictions caused by the first wave of COVID-19.

The participants were adults over 18 years, residents in Madrid (Spain), and in Marrakech and El Jadida (Morocco). An online structured questionnaire using the Google Forms web survey platform with closed questions was created, evaluating sociodemographic variables, eating habits before and during lockdown, habits and lifestyle, anthropometric indicators, and COVID Health.

In June 2020, the questionnaire was sent to students, teachers, and the administration and services staff of the University Autónoma de Madrid, the University Cadi Ayyad de Marrakech, and the University Chouaib Doukkali de El Jadida, who in turn distributed it to at least 10 people in their close social circle. All participants agreed to participate before starting to complete the survey.

The protocol was approved by the Research Ethics Committee of the Autonomous University of Madrid (Ref: CEI-106-2082).

### 2.1. Sample

A non-probabilistic sampling was used in this study. A total of 2302 people (1232 from Spain and 1070 from Morocco) 18 years of age or older responded to the questionnaire: 1530 women (72.56%), 768 men (27.19%), and 4 individuals who did not specify their gender (0.25%). Those who agreed to participate in the study completed an online structured questionnaire using the Google Forms web survey platform.

### 2.2. Variables

Information on the following variables was collected in the survey:

#### 2.2.1. Sociodemographics

Sex, age, educational level (primary, secondary, middle, and higher studies), civil state (single, married, divorced, widow).

#### 2.2.2. Eating Habits Before and During Lockdown

Frequency of daily or weekly consumption of vegetables, fruit, legumes, nuts, meat, fish, eggs, milk, yogurt, cheese, industrial pastries, salty snacks, fast food, and soft drinks before and during lockdown. The questionnaire was based on the validated FFQ in a sample with similar characteristics [24,31].

The frequency of food consumption variable was categorized into five: 0: Never; 1: 1–2 times a week; 2: 3–4 times a week; 3: 5–6 times a week; 4: once a day; and 5: Two or more times a day.

#### 2.2.3. Changes in Eating Behavior During Lockdown

Study participants were asked about the frequency of consumption of certain foods during lockdown by asking the question, “Did you consume with the same frequency, more or less, vegetables, fruits, legumes, nuts, meat, fish, eggs, milk, yogurt, cheese, industrial pastries, salty snacks, soft drinks, wine, and beer and fast food?”

#### 2.2.4. Anthropometric Measures

Self-reported weight (kg) and self-reported height (m) were used to calculate the body mass index (BMI = weight (kg)/height^2^ (m)).

#### 2.2.5. COVID Health

It was also asked whether or not the person had suffered from COVID-19 and, if so, whether or not he or she was hospitalized for it.

#### 2.2.6. Food Insecurity

The scale proposed by Coates’ team [8] was used to calculate Household Food Insecurity Access Scale (HFIAS) for the measurement of food access.

The Food Insecurity Scale based on Experience (FIES-SM) survey module consists of nine questions with dichotomous yes/no answers. The FIES items make up a statistical scale designed to cover a range of severity of food insecurity and should be analyzed together as a scale, not as separate items. The HFIAS occurrence questions relate to three different domains of food insecurity (access) found to be common to the cultures examined in a cross-country literature review [32,33]. The generic occurrence questions, grouped by domain, are as follows:

(1) Anxiety and uncertainty about the household food supply: Did you worry that your household would not have enough food?
(2) Insufficient Quality (includes variety and preferences of the type of food):

Were you or any household member not able to eat the kinds of foods you preferred because of a lack of resources?

Did you or any household member have to eat a limited variety of foods due to a lack of resources?

Did you or any household member have to eat some foods that you really did not want to eat because of a lack of resources to obtain other types of food?

(3) Insufficient food intake and its physical consequences:

Did you or any household member have to eat a smaller meal than you felt you needed because there was not enough food?

Did you or any household member have to eat fewer meals in a day because there was not enough food?

Was there ever no food to eat of any kind in your household because of a lack of resources to get food?

Did you or any household member go to sleep at night hungry because there was not enough food?

Household Food Insecurity Access Scale Indicator Guide. Did you or any household member go a whole day and night without eating anything because there was not enough food?

#### 2.2.7. Statistics Analysis

The data were analyzed using IBM SPSS Statistics version 28.0. Associations between qualitative variables were assessed using the Chi-square test (χ^2^). Differences in food consumption frequencies and levels of food insecurity between the two countries were evaluated using the Student’s *t*-test (for normally distributed continuous variables), the Mann–Whitney U test (for ordinal or non-normally distributed variables), and Chi-square tests (for categorical variables).

To assess the joint impact of sociodemographic variables (country, sex, age, marital status, and educational level) on the probability of experiencing food insecurity, a binary logistic regression was performed, with food insecurity (yes/no) as the dependent variable.

A Multiple Correspondence Analysis (MCA) was also conducted to graphically explore the association patterns between food insecurity categories and food consumption changes during lockdown in both countries.

Normality assumptions were checked using Shapiro-Wilk tests and graphical methods (Q-Q plots and histograms) prior to selecting the appropriate statistical tests.

Given the total sample size and the consistent statistically significant results observed, the estimated statistical power to detect moderate effect sizes was greater than 0.90. Despite the use of a non-probabilistic sampling method, the large sample and analytical strategy support the robustness of the findings. This approach is consistent with methodological arguments presented by [34] regarding the validity of large non-random samples in social research [35].

A significance level of *p* < 0.05 was considered for all analyses.

## 3. Results

The description of the sample is included in Table 1. Table 2 shows that the average HFIAS was higher in the Moroccan sample (3.55) than in the Spanish sample (0.53). The table reveals higher proportions of people suffering from food insecurity in its three forms, mild, moderate, and severe, in Morocco than in Spain.

The details of responses to the HFIAS questions in Table 3 show indeed, that although in both study countries the percentages of people suffering from food insecurity were low in comparison to those of food secure people, the proportions of people in the Moroccan sample declaring feeling worried about obtaining food, or those having a poor diet or forced to reduce or stop consuming foods or favorite foods or having to reduce their dietary diversity or even being forced to skip a meal or to eating for a day due to economic constraints represented significantly higher rates than in Spain.

**Figure 1:**
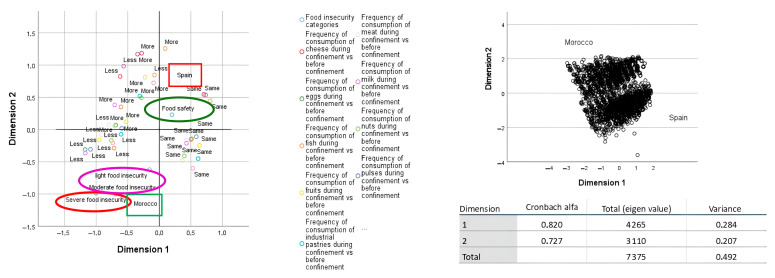
Relationship between changes in food consumption frequency during COVID-19 lockdown and severity of food insecurity in the two samples.

Table 4 shows the changes in dietary habits of the populations studied. During the lockdown, in Morocco, the percentage of people consuming less fruits and fish, less milk, eggs, and those who eat more pulses and more meat was higher than in Spain. But regarding the degree of food insecurity severity, it was observed that those with moderate and severe food insecurity, primarily Moroccans, decreased their consumption of meat, fish, eggs, nuts, legumes, and fruits. Also observed in the Moroccan sample was an increase in the consumption of soda and processed baked goods among those with severe food insecurity (Figure 1).

Factorial Multiple Correspondence Analysis (MCA) is particularly useful for visualizing and exploring relationships between multiple qualitative variables.

In our study, an MCA was conducted to examine the associations between food group consumption patterns and levels of food insecurity. The graphical representation displays the two dimensions that account for the highest variability in the data—together explaining 49.2% of the total variation observed among the variable categories.

As shown in the MCA plot (Figure 1), the frequency of consumption of certain food groups varied systematically with the degree of food insecurity. Individuals experiencing severe food insecurity, primarily those from the Moroccan sample, were more likely to reduce their intake of nutrient-rich foods such as fish, dairy, and fruits, whereas others with lower levels of insecurity showed either no change or an increase in consumption. Moreover, the graph highlights a clear contrast between the two countries studied: Spain is located to the right and Morocco to the left of the first dimension, indicating notable differences in both the severity of food insecurity and the related food behavior changes during the pandemic.

In the Spanish sample and the Moroccan subsample that reported food security, meat and fish consumption remained at the same levels as before the lockdown. Notably, in the Moroccan sample that did not suffer from food insecurity, an increase in the consumption of legumes, nuts, and fruits was observed, and also a higher consumption of soda.

Table 5 shows the results of a multiple logistic regression where food insecurity (no/yes) was the dependent variable and sociodemographic factors—country (Spain/Morocco), gender (Female/Male), marital status (Single, Married—Stable Couple, Separated, Widowed), and level of education attained (Primary, Secondary, Middle, Higher)—were the independent variables.

The risk of insecurity was more than 6 times higher in Morocco than in Spain, more than 13.33 times in men compared to women; 18.55 times higher in separated people with respect to single people; and 16.08 and 14.22 times greater in people with secondary and middle education level compared to university level. Age was a ‘protection factor’ showing a coefficient with a negative sign which indicates a lower risk of food insecurity in less young people.

## 4. Discussion

This article, using the food insecurity index (HFIAS), compares the impact that the lockdown decreed due to the COVID-19 pandemic had on eating behavior in two samples of the university environment from two countries in the Mediterranean basin, Morocco and Spain. Both the average value of the index and the percentage of people in high levels of insecurity present worse values in the Moroccan sample than in the Spanish sample. In both samples, a change is observed in the consumption of some foods compared to the consumption before the pandemic. In the Moroccan sample, changes in eating habits were also observed with less consumption of sodas, salty snacks, and industrial pastries foods likely to have a negative effect on health. On the other hand, they ate less fruit, less fish, milk and eggs, and more legumes and meat than before the lockdown. These results concerning the food consumed during lockdown agree with the Moroccan participants’ statements (Table 3) reporting a poor diet, being forced to reduce or stop the consumption of certain foods in general or their favorite foods, or even reducing their dietary diversity, skipping a meal, or not eating for a day due to economic constraints. These reports concerned significantly higher proportions of Moroccan participants than Spanish participants and are consistent with the differences in rates of food insecurity observed in the two countries. These data corroborate those in the literature reporting an association between household food insecurity and a low quantity of food but also with poor diet quality [36,37,38,39]. This is linked to the negative effect of the pandemic on the Moroccan economy, which resulted in a loss of income for households. This economic situation as well as the imposed lockdown are factors influencing physical accessibility to food or food products as well as household consumption both in quantity and in food quality [22,24,40].

In the Middle East and North Africa (MENA) region, FI was estimated in the adult population at 9.5% between 2014 and 2015 by FAO, with an increase over the following 5 years [41]. In North Africa, this prevalence is estimated at 19.9% and 8.7%, respectively [21]. It is worth noting that the proportion of individuals with severe food insecurity in the Moroccan sample was lower than what is typically reported for the MENA region. This is likely due to the specific characteristics of our sample, which had a medium-to-high level of education—higher than the national average in Morocco. This socioeconomic factor may have contributed to greater dietary resilience despite certain access limitations.

Women and older people were precisely the two groups that started from a better situation. It could be said, therefore, that the economic crisis and the COVID crisis led to a more significant improvement for the people who were already eating well. This leads us to think that the cultural background of the groups with a more developed food culture, women and the elderly, are those who have more experience with regard to culinary activity [42] and who show greater resilience in the face of the vicissitudes of the crisis, as well as taking greater care over their food in difficult times.

The higher prevalence of food insecurity observed in Morocco compared to Spain during the COVID-19 lockdown can be attributed to a combination of pre-existing structural vulnerabilities and differences in the scope and efficacy of governmental response measures. The reasons for the differences observed between the two samples are probably related, firstly, to a starting economic situation, more favorable in Spain than in Morocco, and then to the protection measures for the most vulnerable population taken by the Spanish government. Morocco implemented a rapidly scaled-up emergency response through its *Régime d’Assistance Médicale* (RAMED), extending coverage to nearly 17 million people, particularly targeting informal workers and vulnerable groups. Notably, these transfers reached groups typically excluded from formal social protection mechanisms, such as informal workers, street children, people with disabilities, and the elderly [43]. Despite these efforts, structural limitations in critical care infrastructure and human resources posed significant challenges. Nonetheless, Morocco’s national COVID-19 vaccination campaign was among the most efficient on the African continent, benefitting from early agreements with vaccine suppliers and high public uptake [44].

Spain, on the other hand, leveraged its universal public health and social protection systems. The Spanish government introduced temporary employment regulation schemes (ERTEs), which protected workers’ wages while reducing labor costs for employers. COVID-19 was formally recognized as an occupational disease, thereby enhancing access to paid sick leave and unemployment benefits. Spain’s pre-existing welfare infrastructure enabled rapid deployment of resources, although disparities in coverage and implementation existed, especially for temporary or informal workers.

It is true that in Spain there was a percentage of the population that was faced with a lack of food, loss of work, and resources to cover their basic needs [45,46,47]. In a short time, COVID-19 has magnified already existing disparities in access to food in general and to healthy food in particular in low-income individuals and households [48,49].

In the sample studied, with a medium-high level of education and a stable employment situation, the impact of this situation was low. These results coincide with those obtained for other samples from European Mediterranean countries [50,51]. The results even show an improvement in eating behavior in the Spanish sample, that is undoubtedly associated with being able to stay longer at home and dedicate more time to preparing homemade food while reducing the consumption of fast food and alcohol due to decreased social relationships outside the home [48].

In Morocco, food habits during the lockdown were largely shaped by a return to traditional dietary practices, including the Mediterranean diet and the use of medicinal plants (over 20 species were identified as part of immune-boosting regimens). Households reported a shift towards home cooking, increased consumption of local and organic products, and reliance on herbal remedies. Religious fasting periods (e.g., Ramadan) also influenced food behavior. Cultural resilience and traditional knowledge played a crucial role in maintaining nutritional quality during times of restricted access [52].

We must not forget the results of other Spanish samples with less education level and living in precarious conditions for whom the pandemic resulted in a loss or reduction in work activity and consequently in their choice of food, and even in a greater reliance on charities [53,54,55]. The present research may contribute to the planning and organization of action proposals for situations similar to what occurred in the COVID-19 pandemic. One limitation of this study is that the selected samples are not representative of the general populations from which they originate, as participants had a medium-to-high level of education. Nevertheless, the findings are highly relevant and may provide valuable insights for institutions seeking to develop strategies to address crises that affect access to food, regardless of their origin. Additionally, the results highlight an interesting trend toward healthier eating behaviors and greater adherence to the Mediterranean Diet model. This may be partly explained by the fact that, during periods when individuals have more time available—such as during lockdowns—they are more likely to prepare home-cooked meals, including traditional recipes based on Mediterranean Diet principles. The findings, though general in nature, highlight meaningful disparities in food insecurity between two culturally and economically different Mediterranean countries, and identify key sociodemographic risk factors. These insights can inform more comprehensive future studies that incorporate theoretical frameworks and longitudinal designs to explore causality and longer-term impacts.

## 5. Conclusions

The degree of food insecurity during lockdown in the first wave of the COVID-19 pandemic was much more important in the Moroccan sample than in the Spanish. The higher prevalence of food insecurity observed in Morocco compared to Spain can be attributed to a combination of pre-existing structural vulnerabilities and differences in the scope and efficacy of governmental response measures.

Food insecurity impacted on quantity and quality of the food consumed during lockdown.

In the Moroccan sample, there was a reduction in the consumption of certain foods, the diversity of foods, or even skipping a meal, or not eating due to economic constraints.

In the Spanish sample, a decrease in fast food and alcohol was observed, as well as a significant improvement in diet.

An interesting trend toward healthier eating behaviors and greater adherence to the Mediterranean Diet model is observed in both samples. This may be partly explained by the fact that, during periods when individuals have more time available, they are more likely to prepare home-cooked meals, including traditional recipes based on Mediterranean Diet principles.

Sociodemographic characteristics influenced the severity of insecurity, with men, individuals with lower levels of education, younger people, and those who were single reporting higher levels of insecurity.

## Figures and Tables

**Table 1:** Description of the Sample.

		Spain *n* (%)	Morocco *n* (%)	Statistical Test Χ^2^
Sex	Female	854 (72.7)	673 (60.2)	Χ^2^ = 39.79 *p* < 0.01
	Male	320 (27.3)	422 (39.8)	
Civil status	Single	426 (36.3)	634 (60.0)	Χ^2^ = 131.11 *p* < 0.01
	Married/Stable partner	646 (55.1)	390 (36.8)	
	Separated	63 (5.4)	26 (2.5)	
	Widower/Widow	37 (3.2)	9 (0.8)	
Education Level	Primary	19 (1.6)	30 (2.8)	Χ^2^ = 185.01 *p* < 0.01
	Secondary	92 (7.9)	22(2.1)	
	High school	266 (22.8)	60 (5.7)	
	University	790 (67.7)	947 (89.4)	
Suffered from COVID-19	Yes	157 (12.8)	8 (0.8)	Χ^2^ = 123.92 *p* < 0.01
	No	1069 (87.2)	1057 (99.2)	

**Table 2:** Food Insecurity Index by Country.

	Spain *n* (%)	Morocco *n* (%)	Statistical Test X^2^/ T-Student/ U Mann–Whitney
Food security	1022 (85.5)	545 (52.1)	Χ^2^ = 358.30 *p* < 0.001
Mild food insecurity	78 (6.7)	143 (13.7)	
Moderate food insecurity	44 (3.8)	197 (18.8)	
Severe food insecurity	24 (2.1)	162 (15.5)	
	Mean (SD)	Mean (SD)	
Food Insecurity Index scores	0.53 (1.92)	3.55 (5.24)	t = −17.75 *p* < 0.001
	Median (P25–P75)	Median (P25–P75)	
	0.00 (0.00–0.00)	1.00 (0.00–5.25)	U = 358600 *p* < 0.001

SD: Standard deviation.

**Table 3:** Items of Food Insecurity by Country.

		Spain	Morocco	Chi-Square Test
		*n* (%)	*n* (%)	
Have you been worried about not being able to feed your family?	Never	1020 (87.6%)	684 (65.5%)	χ^2^ = 164.29 *p* ≤ 0.001
	Rarely	78 (6.7%)	132 (12.6%)	
	Sometimes	53 (4.6%)	186 (17.8%)	
	Often	13 (1.1%)	43 (4.1%)	
Have you or someone in your family stopped eating food because you did not have money to buy it?	Never	1112 (95.1%)	657 (63.2%)	χ^2^ = 354.55 *p* ≤ 0.001
	Rarely	34 (2.9%)	155 (14.9%)	
	Sometimes	18 (1.5%)	173 (16.7%)	
	Often	5 (0.4%)	54 (5.2%)	
Have you or someone in your family eaten little variety of food due to financial difficulties?	Never	1085 (92.9%)	672 (64.7%)	χ^2^ = 273.32 *p* ≤ 0.001
	Rarely	42 (3.6%)	140 (13.5%)	
	Sometimes	36 (3.1%)	167 (16.1%)	
	Often	5 (0.4%)	60 (5.8%)	
Have you or someone in your family eaten food that you do not like due to financial difficulties?	Never	1116 (95.9%)	750 (72.2%)	χ^2^ = 240.85 *p* ≤ 0.001
	Rarely	29 (2.5%)	136 (13.1%)	
	Sometimes	18 (1.5%)	113 (10.9%)	
	Often	1 (0.1%)	97.6% (3.8%)	
Have you or someone in your family had a poor diet due to economic difficulties?	Never	1122 (96.1%)	771(74.2%)	χ^2^ = 218.86 *p* ≤ 0.001
	Rarely	29 (2.5%)	135 (13.0%)	
	Sometimes	13 (1.1%)	101 (9.7%)	
	Often	3 (0.3%)	32 (3.1%)	
Have you or someone in your family had to reduce the number of meals per day due to financial hardship?	Never	1135 (97.6%)	823 (79.1%)	χ^2^ = 190.80 *p* ≤ 0.001
	Rarely	19 (1.6%)	108 (10.4%)	
	Sometimes	6 (0.5%)	87 (8.4%)	
	Often	3 (0.3%)	22 (2.1%)	
Has there been a time when you or your family have not been able to eat anything due to financial difficulties?	Never	1152 (98.5%)	920 (88.5%)	χ^2^ = 93.54 *p* ≤ 0.001
	Rarely	13 (1.1%)	70 (6.7%)	
	Sometimes	4 (0.3%)	40 (3.8%)	
	Often	1 (0.1%)	9 (0.9%)	
Have you or someone in your family gone to bed hungry because of financial difficulties?	Never	1151 (98.7%)	934 (89.8%)	χ^2^ = 85.43 *p* ≤ 0.001
	Rarely	11 (0.9%)	56 (5.4%)	
	Sometimes	2 (0.2%)	38 (3.7%)	
	Often	2(0.2%)	12 (1.2%)	
Have you or someone in your family gone an entire day without eating due to financial difficulties?	Never	1161 (99.3%)	979 (94.1%)	χ^2^ = 50.47 *p* ≤ 0.001
	Rarely	7 (0.6%)	34 (3.3%)	
	Sometimes	0 (0%)	21 (2.0%)	
	Often	1 (0.1%)	6 (0.6%)	

**Table 4:** Change in the frequency of food consumption during lockdown.

During Lockdown, He Consumed These Foods with the Same Frequency, More or Less?		Spain	Morocco	Chi-Square Test
		*n* (%)	*n* (%)	
Fruits	More	408 (35.0%)	417 (39.6%)	χ^2^ = 12.45 *p* = 0.002
	Less	155 (13.3%)	170 (16.1%)	
	Same	603 (51.7%)	467 (44.3%)	
Pulses	More	316 (27.0%)	417 (39.6%)	χ^2^ = 69.92 *p* ≤ 0.001
	Less	128 (10.9%)	170 (16.1%)	
	Same	725 (62.0%)	467 (44.3%)	
Nuts	More	374 (32.7%)	245 (23.2%)	χ^2^ = 24.62 *p* ≤ 0.001
	Less	231 (20.2%)	236 (22.4%)	
	Same	538 (47.1%)	574 (54.4%)	
Meat	More	195 (16.8%)	297 (28.2%)	χ^2^ = 42.14 *p* ≤ 0.001
	Less	159 (13.7%)	140 (13.3%)	
	Same	805 (56.6%)	618 (58.6%)	
Fish	More	236 (20.3%)	186 (17.7%)	χ^2^ = 39.68 *p* ≤ 0.001
	Less	245 (21.0%)	346 (32.9%)	
	Same	684 (58.7%)	521 (49.5%)	
Eggs	More	327 (28.0%)	392 (3.4%)	χ^2^ = 58.20 *p* = 0.000
	Less	74 (6.3%)	129 (12.3%)	
	Same	765 (65.6%)	528 (50.3%)	
Milk, yogurt, cheese	More	259 (22.3%)	249 (23.7%)	χ^2^= 33.73 *p* ≤ 0.001
	Less	102 (8.8%)	173 (16.5%)	
	Same	801 (68.9%)	629 (59.8%)	
Industrial pastries	More	233 (21.1%)	176 (16.8%)	χ^2^ = 6.69 *p* = 0.035
	Less	430 (39.0%)	438 (41.8%)	
	Same	440 (39.9%)	435 (41.5%)	
Salty snacks	More	415 (37.1%)	178 (17.0%)	χ^2^ = 109.79 *p* ≤ 0.001
	Less	312 (27.9%)	390 (37.2%)	
	Same	393 (45.0%)	480 (55.0%)	
Sodas	More	240 (21.6%)	123 (12.0%)	χ^2^ = 52.21 *p* ≤ 0.001
	Less	351 (31.7%)	280 (44.4%)	
	Same	518 (46.7%)	623 (60.7%)	

**Table 5:** Predictive mathematical model of food insecurity.

	B	Wald	df	Sig.	Exp(B)	95% C.I. for EXP(B)	
						Lower Bound	Upper Bound
Country of origin (Spain)	1.83	246.04	1	<0.001	6.241	4.965	7.846
Gender (Female)	0.29	7.21	1	0.007	1.335	1.081	1.648
Marital status		7.21	3	0.065			
Married	−0.07	0.29	1	0.593	0.930	0.713	1.213
Separated	0.62	4.24	1	0.039	1.855	1.031	3.339
Widower	0.47	1.11	1	0.292	1.608	0.665	3.890
Age (years)	−0.02	19.59	1	<0.001	0.979	0.969	0.988
Level of education achieved		8.24	3	0.041			
Level of education achieved (primary)	0.35	1.68	1	0.195	1.414	0.837	2.387
Level of education achieved (secondary)	0.47	3.95	1	0.047	1.608	1.007	2.568
Level of education achieved (middle)	0.35	4.54	1	0.033	1.422	1.028	1.966
Constant	−1.37	66.86	1	<0.001	0.253		

Dependent variable: Insecurity (no/yes). Predictive variables: Country (reference: Spain), Gender (reference: Female), Marital Status (reference: Single), Level of education achieved (reference: University), Age.

## Data Availability

The original contributions presented in this study are included in the article. Further inquiries can be directed to the corresponding author.
